# Regional and Gender-Based Distribution of KRAS Mutations in Metastatic Colorectal Cancer Patients in Turkey: An Observational Study

**DOI:** 10.3390/medicina61040694

**Published:** 2025-04-10

**Authors:** Nurullah Ilhan, Faysal Dane, Erdem Goker, Kazım Uygun, Bülent Orhan, Kerem Okutur, İlkay Tuğba Ünek, Abdurrahman Işıkdoğan, Ahmet Bilici, Nurullah Zengin, Necati Alkış, İdris Yücel, Hatice Odabaş, Berna Ömür Öksüzoğlu, Akif Doğan, Hande Nur Erölmez, Mahmut Gümüş

**Affiliations:** 1 Department of Medical Oncology, Sancaktepe Şehit Prof. Dr. İlhan Varank Training and Research Hospital, University of Health Sciences, 34785 Istanbul, Turkey; drakifd@gmail.com (A.D.); herolmez@gmail.com (H.N.E.); 2 Department of Medical Oncology, Faculty of Medicine, Marmara University, 34865 Istanbul, Turkey; faysaldane2001@yahoo.com; 3 Department of Medical Oncology, Faculty of Medicine, Ege University, 35040 İzmir, Turkey; erdem.goker@ege.edu.tr; 4 Department of Medical Oncology, Faculty of Medicine, Kocaeli University, 41285 Kocaeli, Turkey; kzuygun@hotmail.com; 5 Department of Medical Oncology, Acıbadem Bursa Hospital, 16210 Bursa, Turkey; bulentorhan@gmail.com; 6 Department of Medical Oncology, Samsun Training and Research Hospital, University of Health Sciences, 55090 Samsun, Turkey; keremokutur@gmail.com; 7 Department of Medical Oncology, İzmir Tepecik Training and Research Hospital, University of Health Sciences, 06080 İzmir, Turkey; ilkaytugbaunek@gmail.com; 8 Department of Medical Oncology, Faculty of Medicine, Dicle University, 21280 Diyarbakır, Turkey; drisikdogan@hotmail.com; 9 Department of Medical Oncology, Şişli Hamidiye Etfal Training and Research Hospital, University of Health Sciences, 34096 Istanbul, Turkey; ahmetknower@gmail.com; 10 Department of Medical Oncology, Ankara Numune Training and Research Hospital, University of Health Sciences, 06100 Ankara, Turkey; nurzengin@superonline.com; 11 Department of Medical Oncology, Ankara Dr. Abdurrahman Yurtaslan Oncology Training and Research Hospital, University of Health Sciences, 06200 Ankara, Turkey; necatialkis476@gmail.com (N.A.); bernaoksuzoglu@yahoo.com (B.Ö.Ö.); 12 Department of Medical Oncology, Faculty of Medicine, Ondokuz Mayıs University, 55270 Samsun, Turkey; iyucel@omu.edu.tr; 13 Department of Medical Oncology, Haydarpasa Numune Training Research Hospital, University of Health Sciences, Istanbul 34785, Turkey; odabashatice@yahoo.com; 14 Department of Medical Oncology, Istanbul Dr. Lütfi Kırdar Kartal Training and Research Hospital, Istanbul 34890, Turkey; mgumus@superonline.com

**Keywords:** KRAS mutation, metastatic colorectal cancer, anti-EGFR resistance, predictive biomarkers, codon 12 mutation, codon 13 mutation, G12D, G13D, gender differences, regional variation

## Abstract

*Background and Objectives:* KRAS genes are among the most prominent oncogenes that trigger tumor formation in colorectal cancer (CRC) and serve as predictive biomarkers for resistance to anti-EGFR therapies in metastatic colorectal cancer (mCRC) patients. However, the prevalence and mutation spectrum of the KRAS gene family in mCRC patients in Turkey have not been sufficiently analyzed. This study investigates the frequency and distribution of mutations in the KRAS gene family across different regions of Turkey and examines gender-related variations. *Materials and Methods:* This multicenter observational study included 2458 histologically confirmed mCRC patients collected from 52 centers across Turkey. In a central laboratory, KRAS mutations in codons 12 and 13 were analyzed using polymerase chain reaction (PCR). Statistical analyses were performed using chi-square tests and Monte Carlo simulations, with a significance threshold set at *p* < 0.05. *Results:* Depending on the region, KRAS mutations were detected in 45% of patients, ranging from 39.6% to 47.5%. The mutation rate was significantly higher in female patients (48.8%) compared to male patients (42.6%) (*p* = 0.002). Codon 12 mutations were more frequent than codon 13 mutations. G12D, G12V, and G13D mutations accounted for 80% of all detected mutations. The G12V mutation was prevalent in female patients (*p* = 0.007). Based on region, mutation diversity was similar, and no statistically significant difference was found (*p* > 0.05). *Conclusions:* This large-scale, multicenter study provides the most comprehensive dataset of KRAS mutations in mCRC patients in Turkey. This study revealed regional trends, as well as gender differences. The findings highlight the importance of routine KRAS genotyping in guiding personalized treatment strategies, especially regarding candidate selection for anti-EGFR therapies. Further research is required to elucidate the prognostic and therapeutic implications of specific KRAS mutations.

## 1. Introduction

The RAS oncogene is represented by three primary isoforms—HRAS, KRAS, and NRAS—with KRAS being the most frequently identified in colorectal cancer patients [1]. Nonetheless, mutations in any of these isoforms have the potential to transform normal cells into cancerous ones [1,2]. The KRAS gene plays a pivotal role in the pathogenesis of metastatic colon cancer, functioning as an oncogene that regulates key signaling pathways involved in cell proliferation, apoptosis, migration, fate specification, and differentiation [3]. Given the high prevalence of KRAS gene mutations, they have been identified in various malignancies, including pancreatic cancer (90%), thyroid cancer (55%), lung cancer (35%), and rhabdomyosarcoma (35%) [4]. In metastatic colorectal cancer, KRAS mutations occur in approximately 35–40% of cases in Asia [5], 40–50% in Europe [6], and 35–45% in Latin America [7]. Among patients with metastatic CRC, approximately 40–45% harbor activating mutations in KRAS or NRAS [8]. RAS mutations are identified in up to 50% of sporadic colorectal cancers and 50% of colonic adenomas larger than 1 cm. In contrast, these mutations are rarely observed in adenomas measuring 1 cm or less, suggesting that RAS mutations are acquired later during adenoma progression [9,10]. Research has demonstrated that the presence of KRAS mutations is associated with a more aggressive form of colon cancer, characterized by increased metastatic potential and poorer clinical outcomes [3,11,12]. The differential metastatic patterns observed in colon cancer can be attributed to the distinct biological behaviors of tumors based on their KRAS mutation status, which influences their interaction with the tumor microenvironment and the host immune response [13]. For instance, KRAS mutations are correlated with a higher likelihood of metastasis to the liver and lungs, particularly in left-sided primary tumors [14]. These mutations, particularly at codons 12 and 13, result in amino acid substitutions that impair KRAS’s intrinsic GTPase activity, keeping the protein in a permanently active, GTP-bound state. Specifically, at codon 12, the glycine residue is commonly replaced by aspartate (G12D), valine (G12V), or cysteine (G12C), while at codon 13, the most prevalent mutation is G13D [15]. These substitutions prevent the normal hydrolysis of GTP to GDP, leading to the sustained activation of the RAS-RAF-ERK signaling pathway, thereby contributing to oncogenesis. The RAS gene family encodes small GTPase proteins that act as molecular switches in cellular signal transduction, relaying extracellular growth signals to the nucleus. Under normal physiological conditions, RAS proteins alternate between an inactive GDP-bound state and an active GTP-bound state. However, point mutations in RAS typically impair intrinsic GTPase activity, rendering the protein resistant to GTP hydrolysis. As a result, RAS remains locked in its active GTP-bound conformation, leading to a continuous proliferative stimulus and promoting tumor development. However, gain-of-function alterations—such as mutations in KRAS, BRAF, or PIK3CA or the loss of PTEN—lead to the constitutive activation of the PI3K/AKT and MAPK pathways [16]. Epidermal growth factor receptor (EGFR) inhibitors normally block these pathways to suppress tumor growth. However, when KRAS is mutated, it remains permanently active, sending growth and survival signals independently of EGFR stimulation. As a result, anti-EGFR therapies such as cetuximab and panitumumab become ineffective, leading to primary resistance in metastatic colorectal cancer patients [17]. Additionally, constitutive activation of the PI3K/AKT and MAPK pathways activate transcription factors such as NF-κB and AP-1; these transcription factors upregulate the production of pro-inflammatory cytokines and chemokines, including interleukin-8 (IL-8) and granulocyte–macrophage colony-stimulating factor (GM-CSF). Elevated IL-8 functions as a potent chemoattractant for neutrophils and myeloid-derived suppressor cells (MDSCs), while increased GM-CSF promotes the recruitment and differentiation of monocytes into tumor-associated macrophages (TAMs). The accumulation of these immunosuppressive cell types in the tumor microenvironment suppresses the infiltration and activity of cytotoxic T-lymphocytes, thereby creating a “cold” tumor microenvironment that is less responsive to immune attack [18]. These alterations facilitate epithelial–mesenchymal transition (EMT), enabling cancer cells to invade surrounding tissues, resist anoikis (detachment-induced apoptosis), and survive in circulation, ultimately promoting distant organ colonization [18,19]. Putra et al. investigated the association of PD-L1 expression with KRAS mutations and microsatellite instability (MSI) in colorectal cancer patients. Their findings revealed that PD-L1 expression tended to be higher in KRAS wild-type tumors (30%) compared to KRAS-mutant tumors (11%) [20] Similarly, Puccini et al. analyzed the impact of RAS mutations on the tumor immune microenvironment (TIME) and genomic alterations in colorectal cancer patients with microsatellite instability (MSI) or DNA mismatch repair-deficient (dMMR) tumors [21]. Their results demonstrated that RAS-mutant patients exhibited lower tumor mutational burden (TMB) and lower PD-L1 positivity than RAS wild-type patients. Moreover, pathway enrichment analyses indicated a weaker inflammatory profile in the TIME of RAS-mutant tumors [21]. In a recent study, Hu’s team found that KRAS can upregulate CD47, an antiphagocytic signal exploited by tumor cells, through the PI3K/STAT3 pathway to evade innate immune surveillance [22]. Another study found a significant association between L1CAM expression and tumor invasion and metastasis in KRAS mutant patients. Specifically, L1CAM enhanced cellular migration and invasion through the JNK signaling pathway. This effect was mainly observed in KRAS-mutant cancer cells, highlighting the crucial role of L1CAM in tumor progression [23]. Additionally, it has been reported that the L1 cell adhesion molecule (L1CAM) is overexpressed in patients with metastatic colorectal cancer, suggesting its possible role in tumor aggressiveness; however, further studies are needed to fully elucidate its mechanistic relationship with KRAS mutations and its potential as a therapeutic target [24]. In summary, the KRAS gene is intricately linked to the development and progression of metastatic colon cancer. Its mutations drive oncogenic signaling pathways that promote tumor growth and survival and influence cancer cells’ metastatic behavior. The clinical implications of KRAS mutations are profound, as they inform treatment strategies and prognostic assessments for patients with colorectal cancer. Continued research into how KRAS mutations affect tumor biology and the metastatic process is essential for developing more effective therapeutic strategies. KRAS mutation analysis is routinely performed in all metastatic colorectal cancer patients in Turkey. Mapping the geographical distribution of KRAS mutations and their types according to gender across regions may provide a valuable framework for optimizing patient management and establishing region-specific treatment guidelines. Therefore, we conducted a prospective, multicenter, epidemiological observational study over one year to determine the geographical distribution of KRAS mutations and their types by gender in metastatic colorectal cancer (mCRC) throughout Turkey.

## 2. Materials and Methods

### 2.1. Patient Selection and Tissue Samples

This study was conducted across 52 centers, strategically selected to reflect Turkey’s territorial demographic distribution. Following Ethics Committee approval, patients aged 18 years and above with a pathological diagnosis of metastatic colorectal cancer (T1–4, N0–2, M1) who provided written informed consent were enrolled for one year. Patients for whom adequate pathological specimens (tissue samples from either the primary tumor or metastatic sites) could not be collected were excluded from the study. All samples for the KRAS examination were evaluated by an accredited, independent central laboratory in Turkey, and data were recorded in a centralized database to ensure reliability. Tissue samples obtained during routine clinical management were delivered to the Istanbul Genetics Group Genetic Diagnosis Center (Istanbul, Turkey) under appropriate conditions. The available pathological specimens included both primary tumor and metastatic site samples; however, the dataset did not classify specific information regarding the origin of each biopsy (primary vs. metastatic). Sex and the patients’ geographical regions of residence were systematically recorded as part of the study design. The analysis focused on KRAS mutations without incorporating additional demographic or clinical variables such as patient age, smoking status, comorbidities, or specific metastatic organ involvement.

### 2.2. DNA Isolation

Tumor tissues embedded in formalin-fixed paraffin-embedded (FFPE) blocks were initially examined using hematoxylin and eosin (HE) staining, and the tumor area was determined on slides. Then, 6–10 µm thick sections were micro-dissected from these blocks, specifically from tumor-cell-concentrated areas. According to the manufacturer’s instructions, genomic DNA was extracted using the QIAamp DNA FFPE Tissue Kit (Qiagen, Hilden, Germany; Cat. No. 56404). Micro-dissected tissue sections were deparaffinized in xylene and washed with absolute ethanol (both from Merck, Darmstadt, Germany) and air-dried. Cell lysis was carried out using Purgene Cell Lysis Solution (Qiagen, Hilden, Germany; Cat. No. 158116) and proteinase K (Qiagen, Hilden, Germany), incubated at 56 °C overnight. After protein precipitation, DNA was isolated using isopropanol precipitation followed by high-speed centrifugation. The DNA pellet was washed with ethanol, air-dried, and diluted in TE buffer.

The extracted DNA’s quality and quantity were assessed using a NanoDrop 2000 spectrophotometer (Thermo Fisher Scientific, Waltham, MA, USA). PCR amplification of KRAS codons 12 and 13 was performed using a multiplex nested polymerase chain reaction (PCR) protocol on a Veriti Thermal Cycler (Applied Biosystems, Foster City, CA, USA). Following amplification, enzymatic purification was performed with shrimp alkaline phosphatase and exonuclease I (both from New England Biolabs, Ipswich, MA, USA). Mutation detection was carried out using a mini-sequencing technique followed by capillary electrophoresis using a 3130xl Genetic Analyzer (Applied Biosystems, Foster City, CA, USA). Electropherogram peaks were interpreted by comparing the wild-type and mutant alleles corresponding to each nucleotide position within codons 12 and 13.

### 2.3. Statistical Analysis

All statistical analyses were conducted using IBM SPSS Statistics 18 (formerly PASW 18) for Windows. Descriptive statistics are presented as frequencies and percentages for categorical variables and as means, standard deviations, and minimum and maximum values for continuous variables.

The chi-square test was utilized to assess relationships between independent categorical variables. For paired comparisons, Fisher’s Exact Test was employed in cases where the expected frequencies in contingency tables were too small to meet the assumptions of the chi-square test. For multiple group comparisons, Monte Carlo simulation test statistics were applied to ensure robust analysis when chi-square conditions were not fully satisfied.

Subgroup analyses, such as gender and regional comparisons of mutation frequencies, were also performed using these statistical tools. The significance level was set at *p* < 0.05 for all analyses. Where applicable, 95% confidence intervals (CIs) were calculated to provide additional context for significant findings.

This rigorous approach ensured that statistical testing accounted for potential limitations, such as small sample sizes within subgroups, and allowed for accurate comparisons across demographic and clinical variables.

## 3. Results

The demographic and geographical characteristics of the study participants and the distribution of KRAS mutation types are presented below.

Among the 2458 patients included in the study, 61.2% were male (n = 1504), and 38.8% were female (n = 954). Geographically, patients were distributed across five regions in Turkey: Mediterranean and Southeastern Anatolia (11.9%), the Aegean (13.2%), Central Anatolia (21.6%), the Black Sea (10.9%), and Marmara (42.5%). Regarding KRAS mutation status, 45% of patients (n = 1107) had mutant KRAS genes, while 55% (n = 1351) had the wild-type gene, indicating that KRAS mutations were prevalent in nearly half of the mCRC cases (Table 1).

KRAS mutation frequencies based on gender and geographical regions are shown in Table 2. It highlights a significant gender disparity, with 48.8% of female patients having KRAS mutations compared to 42.6% of males (*p* = 0.002). Geographical analysis showed that the frequency of KRAS mutations varied slightly across regions, ranging from 39.6% in the Mediterranean and Southeastern Anatolia region to 47.5% in Central Anatolia. However, these regional differences were not statistically significant (*p* = 0.077), suggesting that KRAS mutation prevalence is relatively uniform across Turkey (Table 2).

Gender-specific KRAS mutation rates within each geographical region are detailed in Table 3. Notably, the Aegean and Central Anatolia regions showed statistically significant differences in mutation frequencies between genders. In the Aegean region, 54.7% of female patients had KRAS mutations compared to 42.3% of males (*p* = 0.030). Similarly, 53.9% of women in Central Anatolia had KRAS mutations compared to 43.5% of men (*p* = 0.020). In the other regions—including Mediterranean and Southeastern Anatolia, the Black Sea, and Marmara—no significant gender differences were observed, with mutation frequencies being similar between men and women (Table 3).

Mutations in codon 12 were more frequent than those in codon 13 among female and male patients (360 and 492 patients, respectively, versus 106 and 149 patients). The most common mutations observed were G12D (n = 341), G12V (n = 308), and G13D (n = 240), collectively accounting for approximately 80% of all KRAS mutations detected in the 1107 patients with mutant KRAS. Notably, the frequency of the G12V mutation—resulting in a glycine-to-valine substitution at codon 12—was significantly higher in female patients than male patients (*p* = 0.007). All other codon 12 and 13 mutations were observed at similar frequencies in both genders (Table 4).

The geographical distribution of specific codon 12 and 13 mutations was consistent across regions, with G12D, G12V, and G13D being the most common alterations. Although slight variations in mutation frequencies were observed, these differences were not statistically significant (*p* > 0.05). For example, the frequency of the G12V mutation ranged from 11.4% in the Aegean region to 13.6% in Central Anatolia. The G13D mutation varied from 6.1% in Mediterranean and Southeastern Anatolia to 11.7% in Central Anatolia. This consistency suggests that the distribution of KRAS mutation types is uniform across different regions of Turkey, irrespective of geographical or environmental factors (Table 5).

## 4. Discussion

This multicenter, epidemiological observational study represents the most extensive investigation of the frequency, anatomical distribution, and mutation spectrum of KRAS mutations in metastatic colorectal cancer (mCRC) patients across Turkey. It has been shown that the frequency of KRAS mutations differs between populations—31.9% in Brazil [25], 13% in Pakistani patients [26], 37% in the Netherlands [6], 17.6% in Albania [27], 39.3% in Germany [28], and 38.5% in China [29]. Previous Turkish studies have highlighted the clinical significance of KRAS mutation testing. In particular, Uçar et al. [30] demonstrated that the number of KRAS mutations in metastatic colorectal cancer (mCRC) is significantly associated with worse clinical outcomes, supporting the role of KRAS as a negative prognostic biomarker. In another study on Turkish CRC patients, researchers analyzed KRAS and BRAF mutation status in a cohort of 220 individuals. The findings indicated that 66.8% of patients had a wild-type KRAS genotype, while 33.2% carried a KRAS mutation in one of the three examined codons (codons 12, 13, and 61) [31]. A study by Gorukmez et al. [32] investigated the distribution of KRAS and BRAF mutations in Turkish patients with metastatic colorectal cancer (mCRC). KRAS mutations were identified in approximately one-third (30%) of cases, with mutations predominantly occurring at codon 12 (73.3%), followed by codon 13 (20%) and codon 61 (6.67%) (34). Ozen et al. [33] reported KRAS mutations in 49.05% of Turkish CRC patients, with codon 12 (65.38%) being the most frequently mutated, followed by codon 13 (26.93%) and codon 61 (7.69%). The remaining 50.95% of cases had wild-type KRAS status. Our study found that 1107 of 2458 mCRC patients (45%) harbored mutant KRAS genes, with regional frequencies ranging from 39.6% to 47.5%, and also detected a significant gender disparity, with female patients exhibiting a higher frequency of KRAS mutations than male patients (48.8% vs. 42.6%; *p* = 0.002). Similar findings have been reported in previous Turkish cohorts [30,33]. Similarly, a study of 8234 Brazilian mCRC patients reported significantly higher KRAS mutation rates in females [25]. In some studies, the high frequency of mutant KRAS in female patients has been attributed to hormonal factors, particularly estrogen [34,35]. Lingeng Lu et al., using a 3D organoid model with *KRAS* mutant (MT) and *KRAS* wild-type (WT) CRC cells [34], demonstrated that E2 supplementation had no effect on *KRAS* MT cells when grown in a glutamine-depleted medium but significantly inhibited WT cells. These results suggest that CRC growth depends on tumor nutrient availability, *KRAS* mutation status, and E2; E2 exposure decreases CRC cell growth but has no significant effect when the cells harbor *KRAS* mutations and are cultured under nutrient-depleted conditions. Similarly, Topi et al. (2022) [35] demonstrated a correlation between ERα (Estrogen Receptor Alpha) and ERβ (Estrogen Receptor Beta) expression and KRAS mutation status. In this study, KRAS-mutant colorectal cancer patients exhibited higher ERα expression levels, whereas ERβ expression tended to be lower than wild-type KRAS patients. Additionally, KRAS mutations were more frequently observed in patients with positive ERα expression. At the same time, an inverse trend was noted for ERβ expression, with KRAS mutations less prevalent in patients exhibiting high ERβ expression. These findings suggest a potential interplay between estrogen receptor signaling and KRAS-driven colorectal cancer pathogenesis, where ERα upregulation may be associated with KRAS mutations [35]. Although direct evidence linking estrogen to a higher frequency of KRAS mutations in colorectal cancer remains limited, these mechanistic insights provide a plausible explanation for the observed gender differences. In our study, the marked difference in KRAS mutation frequency between women and men in regions such as the Aegean and Central Anatolia suggests that, in addition to estrogen, other factors may also contribute to this disparity. One study demonstrated that rectal and distal colon tumors are more likely to harbor specific KRAS mutations compared to proximal colon cancers [36]. Moreover, variations in environmental exposures and lifestyle factors—such as dietary habits, smoking rates, and physical activity—have been linked to colorectal carcinogenesis and may further modulate the genetic mutation spectrum [37,38]. These findings imply that in regions like the Aegean and Central Anatolia, the interplay between tumor anatomical site and local environmental/lifestyle factors could drive the higher KRAS mutation frequencies observed in female patients. Further investigation is needed to clarify these complex interactions.

Significantly, our study’s inclusion of a large, geographically diverse Turkish cohort, combined with robust central laboratory analysis, mitigates the variability observed in previous studies and provides a more reliable estimate of the KRAS mutation spectrum in Turkish mCRC patients. In the Turkish health system context—where personalized medicine is increasingly emphasized—our findings support the routine implementation of KRAS testing to optimize treatment strategies and improve patient outcomes. International guidelines from NCCN, ASCO, and the European Medicines Evaluation Agency recommend that advanced CRC patients undergo KRAS mutation testing since anti-EGFR therapies such as cetuximab and panitumumab are effective only in patients with wild-type KRAS [39,40]

In previous studies, the predominant mutations in codons 12 and 13 of the KRAS gene—namely, G12D, G12V, and G13D—accounted for approximately 80% of the mutations detected, and similar proportions were observed in our study [41]. Imamura et al., in a study involving 1075 BRAF wild-type cancers, reported that patients harboring KRAS codon 12 mutations exhibited significantly higher colorectal-cancer-specific mortality compared with those with KRAS wild-type/BRAF wild-type tumors. In contrast, KRAS codon 13 mutations were not significantly associated with prognosis [42]. In one study, among the seven most common KRAS mutations, the c.35G>T (p.G12V) mutation was associated with significantly higher colorectal cancer-specific mortality compared to KRAS wild-type/BRAF wild-type cases [42]. These findings suggest that KRAS codon 12 mutations, especially p.G12V, may be prognostically adverse, while codon 13 mutations may not impart a similar negative impact.

However, it is essential to note that not all point mutations in KRAS uniformly predict resistance to anti-EGFR monoclonal antibody therapy. In a pooled analysis of 579 mCRC patients from seven clinical trials, De Roock et al. [43] demonstrated that although KRAS mutations were generally associated with decreased survival, patients with KRAS G13D mutations experienced better overall and progression-free survival following cetuximab treatment compared with other KRAS mutant subtypes. This nuanced understanding underscores the need for mechanistic studies to delineate how specific KRAS mutations influence treatment outcomes precisely.

## 5. Conclusions

In this multicenter study of 2458 metastatic colorectal cancer (mCRC) patients across Turkey, we found that KRAS mutations were present in 45% of cases, with no significant regional variation. However, a gender disparity was observed, as KRAS mutations were significantly more frequent in female patients (48.8%) compared to male patients (42.6%) (*p* = 0.002).

Although regional KRAS mutation frequencies were similar across Turkey (ranging from 39.6% to 47.5%), notable gender-based differences were identified in the Aegean (*p* = 0.030) and Central Anatolia (*p* = 0.020) regions, where female patients had significantly higher mutation rates than male patients.

Among specific KRAS mutations, codon 12 alterations were more common than codon 13 in both male and female patients. The most frequently observed mutations were G12D (n = 341), G12V (n = 308), and G13D (n = 240), accounting for 80% of all KRAS mutations. Interestingly, the G12V mutation was significantly more common in women than men (*p* = 0.007), whereas all other mutations were evenly distributed between genders.

The geographical distribution of specific KRAS mutations (e.g., G12D, G12V, and G13D) was broadly consistent across Turkey, with no statistically significant regional differences (*p* > 0.05). These findings suggest that while KRAS mutations are evenly distributed across different regions, gender-related differences exist, particularly in the specific areas and mutation subtypes.

Our results highlight the importance of considering gender differences and regional variation in KRAS mutational profiling in mCRC patients. The observed geographical differences in gender-specific KRAS mutation rates suggest that genetic, environmental, and lifestyle factors may contribute to these variations. Further research is needed to explore the clinical implications of these differences, particularly regarding treatment response and personalized therapeutic strategies.

## 6. Limitations

This study has several limitations. First, the cross-sectional design precludes the evaluation of treatment outcomes and the prognostic implications of KRAS mutations. Additionally, the analysis was limited to KRAS mutations in codons 12 and 13, excluding other potentially relevant mutations, such as those in the BRAF gene or additional KRAS codons. Furthermore, the dataset did not systematically record whether the analyzed samples originated from primary tumors or metastatic lesions. As a result, we could not perform a comparative analysis between primary and metastatic tumors, which represents a significant limitation in understanding potential differences in KRAS mutation prevalence between these tumor sites. Similarly, the lack of stratification by tumor location (e.g., right-sided versus left-sided colorectal cancer) further limits our understanding of how mutation prevalence may vary based on tumor site. Although this study included regional stratification, other demographic and clinical factors that could influence KRAS mutation distribution, such as patient age, smoking status, comorbidities, and specific metastatic organ involvement, were not collected. Finally, the absence of longitudinal follow-up data prevents insights into patient survival or response to targeted therapies, leaving the clinical impact of specific mutations unexplored. Future studies addressing these limitations are warranted to provide a more comprehensive understanding of KRAS mutations in metastatic colorectal cancer.

## Figures and Tables

**Table 1:** Distribution of study patients by gender, geographical region, and KRAS mutation status. Descriptive statistics are frequency (n) and percentage (%). For group comparisons, chi-square tests were performed.

Variable	Category	n	%
Gender	Female	954	38.8
	Male	1504	61.2
Geographical Region	Mediterranean and Southeastern Anatolia	293	11.9
	Aegean	324	13.2
	Central Anatolia	530	21.6
	Black Sea	267	10.9
	Marmara	1044	42.5
KRAS Mutation Status	Mutant	1107	45.0
	Wild-type	1351	55.0

**Table 2:** Gender and geographical distribution of mutant and wild-type KRAS gene. Data are presented as frequency (n) and percentage (%). *p*-values were generated using chi-square tests.

Variable	Category	Mutant (n = 1107)	Wild (n = 1351)	*p*-Value
Gender	Female	466 (48.8)	488 (51.2)	0.002
	Male	641 (42.6)	863 (57.4)	
Region	Mediterranean and Southeastern Anatolia	116 (39.6)	177 (60.4)	0.077
	Aegean	153 (47.2)	171 (52.8)	
	Central Anatolia	252 (47.5)	278 (52.5)	
	Black Sea	107 (40.1)	160 (59.9)	
	Marmara	479 (45.9)	565 (54.1)	

**Table 3:** Comparison of the percentages of female and male patients with mutant KRAS gene in the regions. Data are shown as frequency (n) and percentage (%); *p*-values were calculated using chi-square tests.

Region	Gender	Mutant (n, %)	Wild-Type (n, %)	*p*-Value
Mediterranean and SE Anatolia	Female	41 (41.0%)	59 (59.0%)	0.722
	Male	75 (38.9%)	118 (61.1%)	
Aegean	Female	70 (54.7%)	58 (45.3%)	0.030
	Male	83 (42.3%)	113 (57.7%)	
Central Anatolia	Female	111 (53.9%)	95 (46.1%)	0.020
	Male	141 (43.5%)	183 (56.5%)	
Black Sea	Female	46 (45.5%)	55 (54.5%)	0.155
	Male	61 (36.7%)	105 (63.3%)	
Marmara	Female	198 (47.3%)	221 (52.7%)	0.466
	Male	281 (45.0%)	344 (55.0%)	

Note: *p*-values represent overall comparisons for each region.

**Table 4:** Comparison of the point mutations in codons 12 and 13 of the KRAS gene between male and female patients. Data are presented as frequency (n) and percentage (%); *p*-values were calculated using chi-square tests.

Point Mutation	Female (n = 954)	Male (n = 1504)	*p*-Value
G12A	10 (1.0%)	21 (1.4%)	0.451
G12C	48 (5.0%)	63 (4.2%)	0.327
G12D	138 (14.5%)	203 (13.5%)	0.499
G12R	8 (0.8%)	11 (0.7%)	0.767
G12S	15 (1.6%)	27 (1.8%)	0.678
G12V	141 (14.8%)	167 (11.1%)	0.007
G13C	2 (0.2%)	7 (0.5%)	0.496
G13D	102 (10.7%)	138 (9.2%)	0.217
G13R	2 (0.2%)	4 (0.3%)	1.000

**Table 5:** Comparison of the point mutations in codons 12 and 13 of the KRAS gene across the regions of Turkey. Data are presented as frequency (n) and percentage (%); *p*-values were calculated using chi-square tests.

Point Mutation	Mediterranean and SE Anatolia (n = 293)	Aegean (n = 324)	Central Anatolia (n = 530)	Black Sea (n = 267)	Marmara (n = 1044)	*p*-Value
G12A	2 (0.7%)	4 (1.2%)	6 (1.1%)	4 (1.5%)	15 (1.4%)	0.877
G12C	14 (4.8%)	18 (5.6%)	26 (4.9%)	13 (4.9%)	40 (3.8%)	0.689
G12D	35 (11.9%)	51 (15.7%)	74 (14.0%)	28 (10.5%)	153 (14.7%)	0.292
G12R	5 (1.7%)	1 (0.3%)	3 (0.6%)	3 (1.1%)	7 (0.7%)	0.270
G12S	7 (2.4%)	5 (1.5%)	7 (1.3%)	2 (0.7%)	21 (2.0%)	0.498
G12V	34 (11.6%)	37 (11.4%)	72 (13.6%)	34 (12.7%)	131 (12.5%)	0.888
G13C	1 (0.3%)	2 (0.6%)	1 (0.2%)	0 (0.0%)	5 (0.5%)	0.717
G13D	18 (6.1%)	33 (10.2%)	62 (11.7%)	23 (8.6%)	104 (10.0%)	0.130
G13R	0 (0.0%)	2 (0.6%)	1 (0.2%)	0 (0.0%)	3 (0.3%)	0.557

## Data Availability

The data presented in this study are available upon request from the corresponding author. Any requests will be reviewed against compliance with ethical, scientific, regulatory, and legal requirements. Requests to access the datasets should be directed to nurullahilhan07@gmail.com.
